# Ab-Externo Implantation of XEN Gel Stent for Refractory Steroid-Induced Glaucoma After Lamellar Keratoplasty

**DOI:** 10.7759/cureus.13320

**Published:** 2021-02-12

**Authors:** Shu Yu Tan, Norshamsiah Md Din, Safinaz Mohd Khialdin, Wan Haslina Wan Abdul Halim, Seng Fai Tang

**Affiliations:** 1 Ophthalmology, Universiti Kebangsaan Malaysia Medical Centre, Kuala Lumpur, MYS

**Keywords:** xen gel stent, minimally invasive glaucoma surgery, migs, ab-externo, steroid induced glaucoma, lamellar keratoplasty

## Abstract

The hazy corneal donor-recipient interface after corneal transplant may cause difficulties when implanting the XEN gel stent via ab-interno approach. We aim to describe XEN gel stent implantation via ab-externo approach in refractory steroid-induced glaucoma after corneal lamellar keratoplasty. Under local anaesthesia, the XEN injector needle was inserted 7 mm behind the limbus with the bevel facing up, directly beneath the conjunctiva and advanced to the marked 2.5 mm scleral entry wound. The needle then pierced the sclera until the needle tip was just visible in the anterior chamber (AC). The slider was pushed until the tip of the XEN stent was seen in the AC. The needle was slowly withdrawn while still pushing the slider to complete stent deployment. Subconjunctival Mitomycin C 0.01% (30 µg/0.3 mL) was then injected posterior to the bleb. Three eyes of three patients with steroid-induced glaucoma after lamellar keratoplasty underwent XEN gel stent implantation via ab-externo approach placed at the superotemporal quadrant. Pre-operatively, all patients had uncontrolled IOP between 30-45 mmHg despite maximum medications and selective laser trabeculoplasty. After XEN gel stent implantation, IOP ranged between 10-17 mmHg with one or two topical antiglaucoma at 12 months. Complications include hypotony maculopathy, stent migration and hyphaema, all of which were successfully managed. Corneal graft remained clear at 12 months. XEN gel stent implantation via ab-externo approach is able to achieve good intraocular pressure (IOP) control without compromising cornea graft in patients with steroid-induced glaucoma after lamellar keratoplasty at 12 months.

## Introduction

Minimally invasive glaucoma surgery (MIGS) is an emerging surgical treatment with good safety profile and rapid recovery. XEN gel stent (Allergan, Irvine, CA, USA) is an MIGS device reported to provide up to 56% of intraocular pressure (IOP) reduction and reduce up to 2.7 antiglaucoma medication at 12 months [1], with lower complication rate compared to conventional trabeculectomy [2].

In corneal transplant cases, implanting XEN gel stent via the recommended ab-interno approach may be difficult given the hazy view of the graft-host interface on the cornea. We describe an ab-externo approach of XEN gel stent implantation in refractory steroid-induced glaucoma after corneal lamellar keratoplasty.

## Technical report

**Figure 1:**
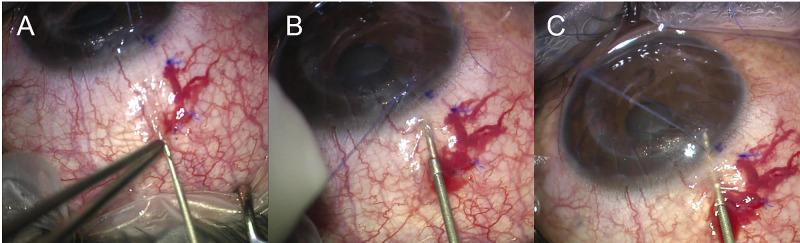
Steps depicting ab-externo XEN implantation. A: Conjunctival insertion at 7 mm behind limbus. B: Scleral insertion at 2.5 mm behind limbus. C: XEN injected when injector tip is seen in the anterior chamber.

All XEN gel stent implantations were performed via ab-externo approach and placed at the superotemporal quadrant. Topical Proparacaine hydrochloride 0.5% (Alcaine, Alcon, Fort Worth, TX, USA) was used for anaesthesia. Because the pre-operative IOP was more than 30 mmHg in all cases, we performed a slow paracentesis to prevent a sudden drop in IOP and limit the size of the bleb after implantation. A high IOP may cause too huge a bleb and shallowing of the anterior chamber (AC). Corneal traction with Polyglactin 7-0 suture (Vicryl, Ethicon, Somerville, NJ, USA) was placed at the intended quadrant. The sites for conjunctival and scleral entry wounds were then marked at 7 mm and 2.5 mm respectively behind the limbus (Figure 1A, 1B). Subconjunctival Lignocaine hydrochloride 2% injection (Lignocaine HCl, Ain Medicare, Malaysia) separates the conjunctiva from tenon and provides further anaesthesia. Over-inflation of the subconjunctival space is avoided to maintain the accuracy of entry point markings. 

With the assistance of a conjunctival forceps, the XEN injector needle pierced the conjunctiva 7 mm behind limbus with the bevel facing up. The needle is then advanced in the subconjunctival space towards the 2.5 mm marking of the scleral entry wound. With the eye placed at primary position and applying countertraction using the corneal traction suture, the needle pierced the sclera at a direction just anterior and parallel to the iris plane, aiming towards the centre of the cornea, until the tip of the needle can be seen in the AC. This manoeuvre is somewhat similar to the direction when a scleral track is created through which the tube of a glaucoma drainage device is inserted. The slider of the Xen injector device was then pushed slowly until the tip of the XEN stent can be seen in the AC (Figure 1C). Once the tip of XEN stent was visible, the needle was gently retracted to avoid inadvertent side push of the injector and premature dislodgement of the XEN stent. The plunger was pushed continuously while slowly withdrawing the needle to prevent too long a portion of the XEN stent in the AC. With this technique, good stent placement can be achieved with 2 mm each in the AC, scleral tunnel and subconjunctival space, and the AC portion of the Xen stent sits between the cornea and the iris. Subconjunctival Mitomycin C 0.01% (30 µg/0.3 mL) (Mitomycin C, Kyowa, Japan) was then injected posterior to the bleb.

Postoperatively, all patients received Dexamethasone 0.1% (Maxidex, Alcon, Fort Worth, TX, USA) and Ciprofloxacin 0.3% (Zoxan, FDC LTD, Mumbai, India) every two hours, and all antiglaucoma medications were discontinued. Patients were seen on day one postoperatively, then weekly in the first month. Antimetabolite injection with 5-fluorouracil 5 mg/0.1 mL (DBL Fluoracil, Hospira, UK) or Mitomycin C 0.01% (30 µg/0.3 mL) and Dexamethasone 0.4 mg/0.1 mL were performed with or without bleb needling when there were signs of subconjunctival fibrosis or high-risk bleb failure [3,4]. Corticosteroid eyedrop was tapered to pre-operative regime after two months for the purpose of corneal graft survival.

Three patients underwent XEN gel stent implantation via ab-externo approach (Table 1). They were on prolonged use of topical corticosteroid, either Loteprednol etabonate 0.5% (Lotemax, Bausch & Lomb, Bridgewater, NJ, USA) or Fluoromethalone 0.1% (FML, Allergan, Madison, NJ, USA) after lamellar keratoplasty. Pre-operatively, all patients had moderate to advanced steroid-induced glaucoma with uncontrolled IOP ranged from 30 to 45 mmHg despite on maximal topical, systemic antiglaucoma agents and selective laser trabeculoplasty (Table 2).

**Table 1:** Patient demographic and preoperative data. DALK: deep anterior lamellar keratoplasty; DSAEK: Descemet stripping automated endothelial keratoplasty; PBK: pseudophakic bullous keratopathy; VCDR: vertical cup-to-disc ratio, MD: mean deviation; HVF: Humphrey visual field.

Case No.	Age (year)	Right/left	Lamellar keratoplasty			Angle	VCDR	MD on HVF (dB)
			Type	Duration (months)	Reason			
1	28	Right	DALK	5	Keratoconus	Open	0.4	-10.76
2	32	Left	DALK	12	Keratoconus	Open	0.7	-14.24
3	83	Right	DSAEK	16	PBK	Open	0.8	-14.72

**Table 2:** Preoperative and postoperative data. IOP: intraocular pressure; CDVA: corrected distance visual acuity.

Case no.	Follow-up (month)	IOP (mm Hg)			No. of antiglaucoma		CDVA		Duration of complete success (months)
		Preop	Postop day 1	Postop 12 months	Preop	Postop	Preop	Postop	
1	12	38	10	17	6	1	6/18	6/18	5
2	12	45	8	17	6	2	6/24	6/18	9
3	12	30	7	10	5	1	6/18	6/12	8

**Figure 2:**
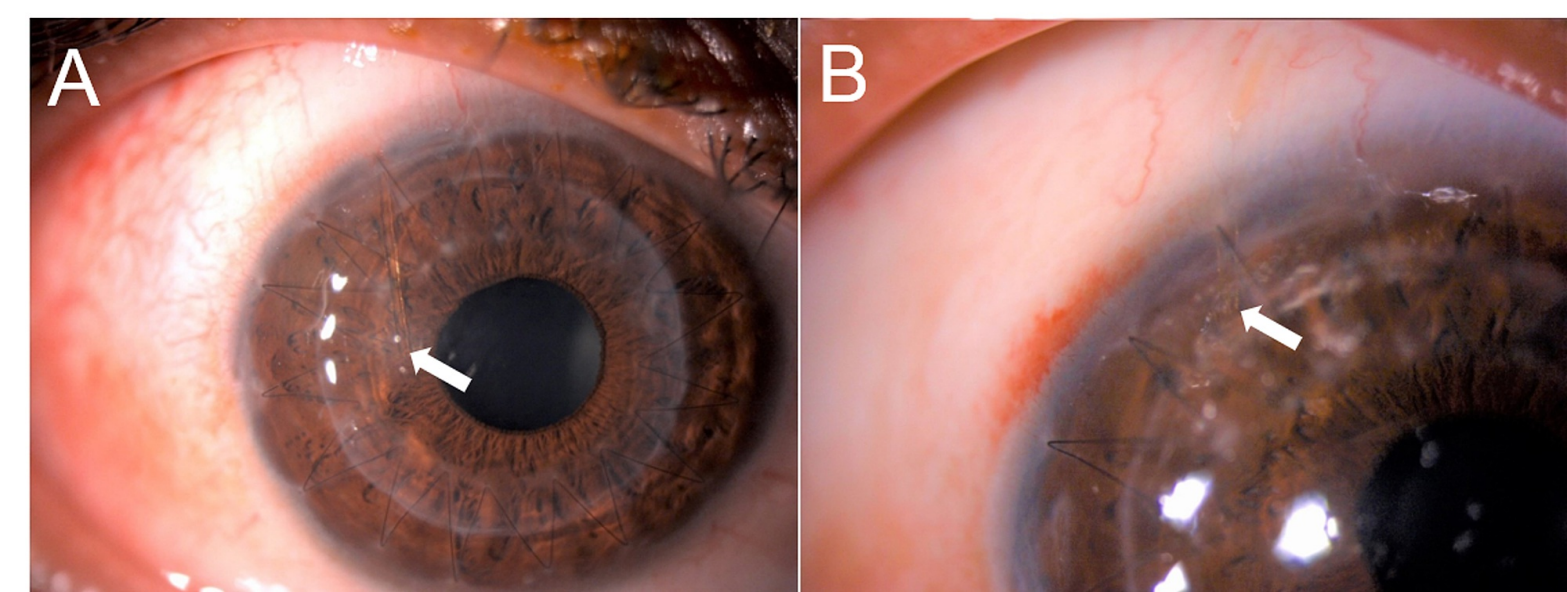
XEN gel stent migration in first case. A: XEN gel stent (arrow) migration towards the anterior chamber on postoperative day two. B: stable XEN gel stent (arrow) after repositioning.

First case was complicated with shallow AC, XEN gel stent migration and hypotony maculopathy. He developed acute gouty arthritis and was struggling to ambulate. Deep AC on day one became shallow on day two with IOP of 3 mmHg and macular striation. He underwent AC reformation with cohesive viscoelastic agent after which, the AC was deep with IOP of 9 mmHg. On day two, the stent progressively migrated into the AC (Figure 2A). It was successfully repositioned using intraocular forceps from within the AC (Figure 2B). His vision recovered to baseline after two months. At five months, IOP peaked at 26 mmHg with early bleb fibrosis. Timolol maleate 0.5% (Iotim, FDC LTD, Mumbai, India) was added with antimetabolite injection and bleb needling every one to two weeks. At nine months, IOP stabilized with diffuse and low-lying bleb.

Second case had transient minimal hyphaema on day one postoperatively which resolved spontaneously. At week two, IOP was 18 mmHg with flat and vascularized bleb. After two needlings, the bleb was diffuse and elevated. His IOP maintained between 12 and 14 mmHg without medication. Nine months later, he developed suture-related fungal corneal ulcer which took three months to heal with the empirical treatment of topical, intracameral and systemic antifungal. During the infection, AC was deep with fibrinous aqueous, bleb was flat and IOP was as high as 58 mmHg, requiring up to three topical and two systemic antiglaucoma. After the infection was under control, bleb needling was performed when it was deemed safe by the corneal specialist. At 12 months, he was on Timolol maleate 0.5% and Brimonidine Tartrate 0.15% (Alphagan P, Allergan, Irvine, CA, USA).

**Figure 3:**
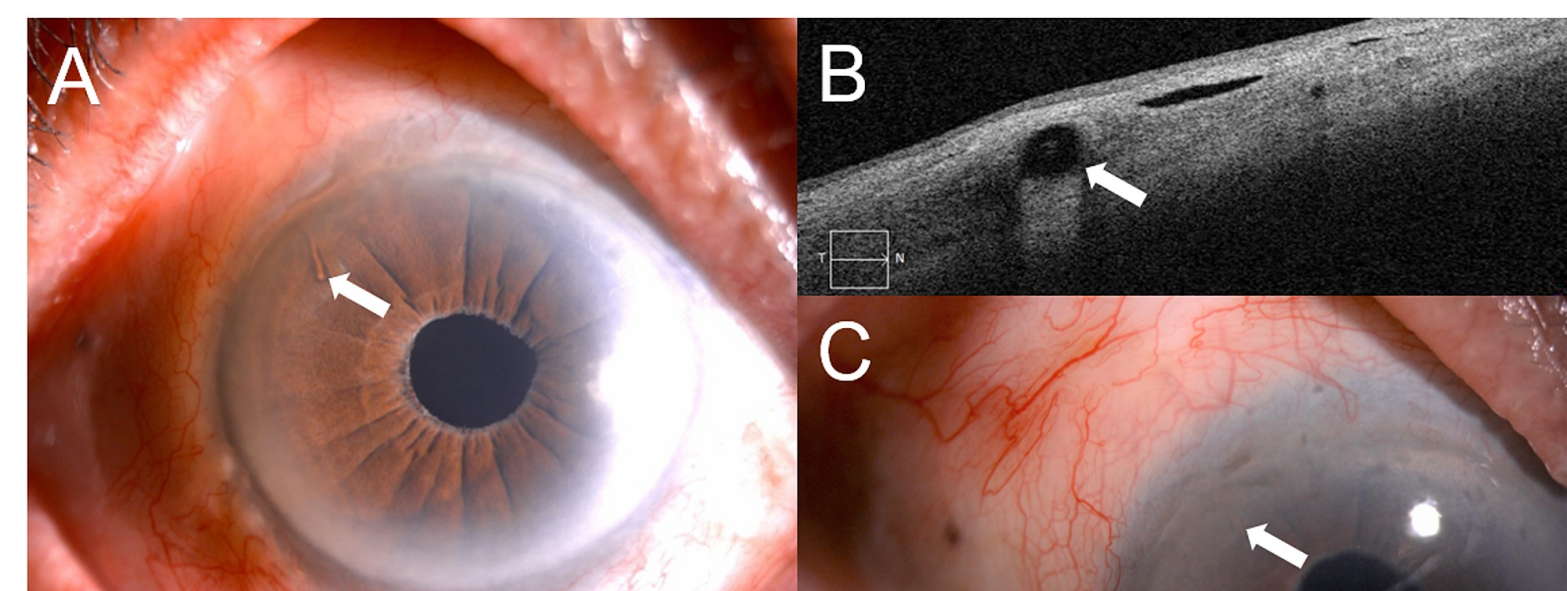
XEN gel stent implantation in third case. A: a well-placed XEN gel stent (arrow) at the superotemporal quadrant. B: anterior segment-optical coherence tomography of the XEN gel stent (arrow) with a diffuse filtration bleb. C: XEN gel stent in situ (arrow) with diffuse, low-lying bleb.

In the third case, early fibrosis was seen in the bleb and needling was performed at week four. The bleb was diffuse (Figure 3A, 3B), with IOP ranging between 6 and 10 mmHg without medication for up to eight months. Subsequently, IOP raised to 28 mmHg with encapsulated bleb which required two antiglaucoma. After two bleb needlings, the bleb was diffuse (Figure 3C) and he required only Latanoprost 0.005% (Xalatan, Pfizer, Belgium).

After 12 months, the total number of antimetabolite injections performed was 14 in first case, six in second case, and five in third case, with an average of four bleb needling done. Bleb was diffuse and low-lying with clear corneal graft in all patients.

## Discussion

XEN gel stent is a porcine-based 6 mm gelatin implant that drains aqueous to the subconjunctival space through a 45 µm lumen. These dimensions provide a steady-state pressure of 6-8 mmHg at 2-2.5 µL/min flow rate, preventing postoperative hypotony [5]. Due to its hydrophilic nature, the XEN stent hydrates and conform to the tissue after implantation, reducing tissue erosion, stent migration and corneal endothelial damage [6].

The recommended technique of XEN gel stent implantation is via ab-interno approach. However, following corneal lamellar keratoplasty, the graft-host interface is often hazy, precluding good view for an ab-interno implantation. An ab-externo technique allows XEN implantation even with poor gonioscopic view from corneal opacity at the graft-host interface. Additionally, there is a low risk of corneal endothelium or lens injury as there are no instruments going into the anterior chamber, especially in phakic young patients with keratoconus like our first two cases. Placement at the superotemporal quadrant reduces the risk of bleb dysesthesia [7]. Further, in an experimental system, ab-interno approach was shown to have greater outflow resistance and less predictable bleb formation than an ab-externo approach [8].

The incidence of high IOP after corneal transplant is approximately 46.5% [9]. Close to 10% of them eventually required glaucoma surgery [9,10]. Steroid-induced glaucoma is especially common after prolonged postoperative steroid used to ensure graft survival. The resultant elevated IOP may cause both graft failure and irreversible glaucomatous optic neuropathy [11]. Multiple antiglaucoma drops may cause epithelial toxicity and threatens graft survival. Laser trabeculoplasty, while potentially successful, is limited by poor angle view and short-lived efficacy [11]. Conventional surgical interventions may reduce the number of medications but are not without risks. Glaucoma drainage devices cause graft failure in 20%-74% of cases because of the proximity of the tube to the corneal endothelium causing mechanical cell loss. While pars plana tube implantation prevents this complication, posterior segment complications are equally detrimental [11].

XEN gel stent, being smaller in calibre and biologically inert, may be gentler to the cornea. The transient and low complication rate such as hypotony (3.3%), hypotony maculopathy (0.3%), flat AC (1.1%) and implant migration (0.3%)1 makes it more favourable. Our first case had hypotony with shallow AC and implant migration, probably from the exertion to mobilize because of acute gouty arthritis causing repetitive AC depth and IOP fluctuation [12,13].

Our second case had suture-related fungal corneal ulcer at nine-month post-operatively. Although this complication is unrelated to the XEN gel stent implantation, his bleb closed down from the intense inflammation during the infection. After the infection was brought under control, bleb needling with Mitomycin C 0.01% managed to re-instate the bleb, proving that there was no stent occlusion as previously reported [14].

Antiglaucoma medication was reduced from an average of 5.6 to 1.3 agents at 12 months. IOP control was good without antiglaucoma for a mean of 7.3 months post-operatively. At 12 months, all three patients had qualified success of XEN gel stent implantation, with one to two topical antiglaucoma and no disease progression, even with continued use of corticosteroid given for corneal graft survival.

## Conclusions

Ab-externo XEN gel stent implantation offers an external approach that eliminates the use of viscoelastic and risk of lens touch. It is effective in the treatment of refractory steroid-induced glaucoma in lamellar keratoplasty patient without compromising the cornea. Complications were transient and the corneal grafts in all patients remained clear after a minimum follow-up of 12 months.
